# Supplementary material for: Combined Transcriptome and Metabolome Analysis Reveals That Carbon Catabolite Repression Governs Growth and Pathogenicity in Verticillium dahliae
Source: Int J Mol Sci. 2024 Oct 28;25(21):11575. doi: 10.3390/ijms252111575 (PMC11546859; doi:10.3390/ijms252111575)

Starch condition

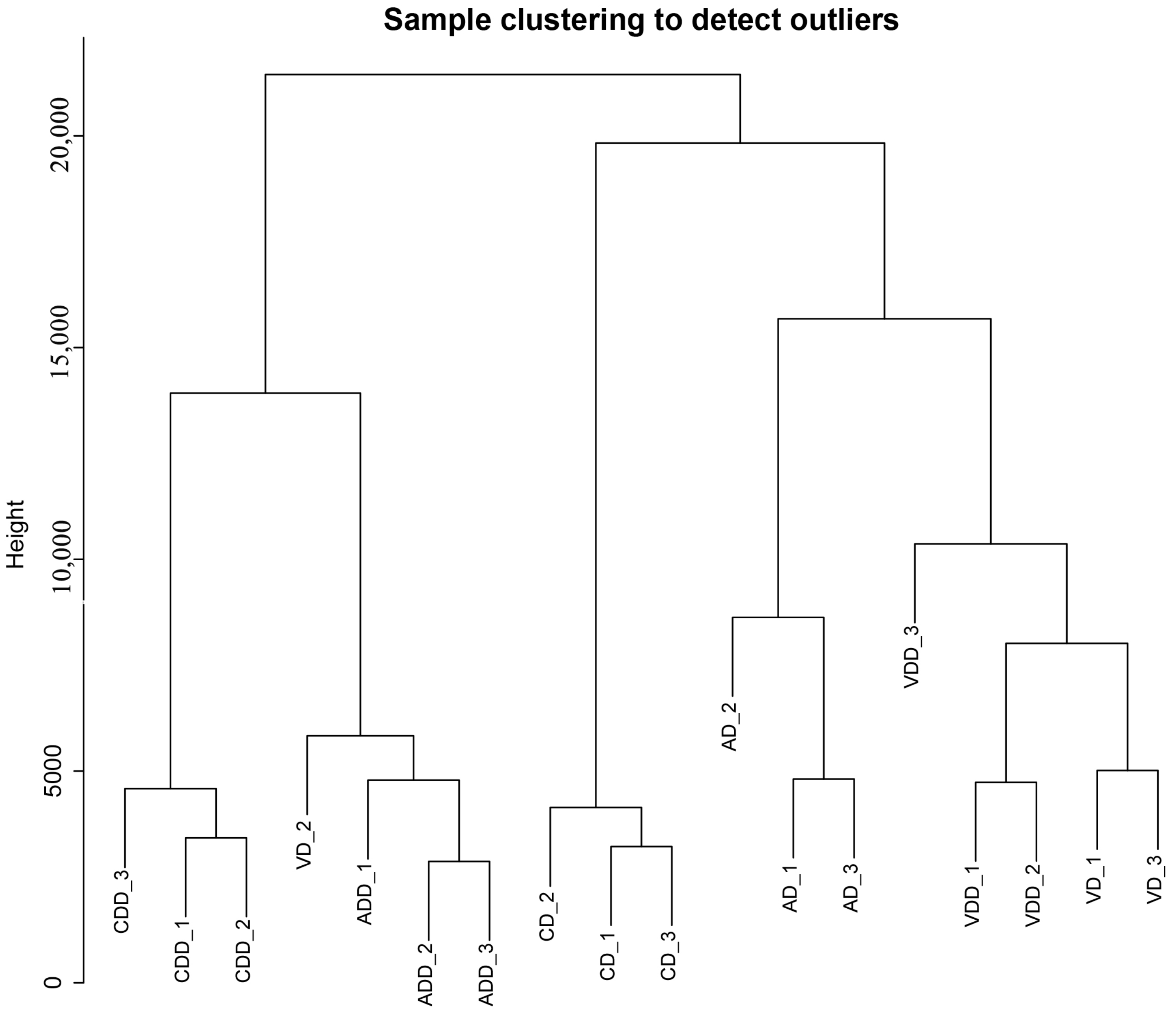

Cellulose condition

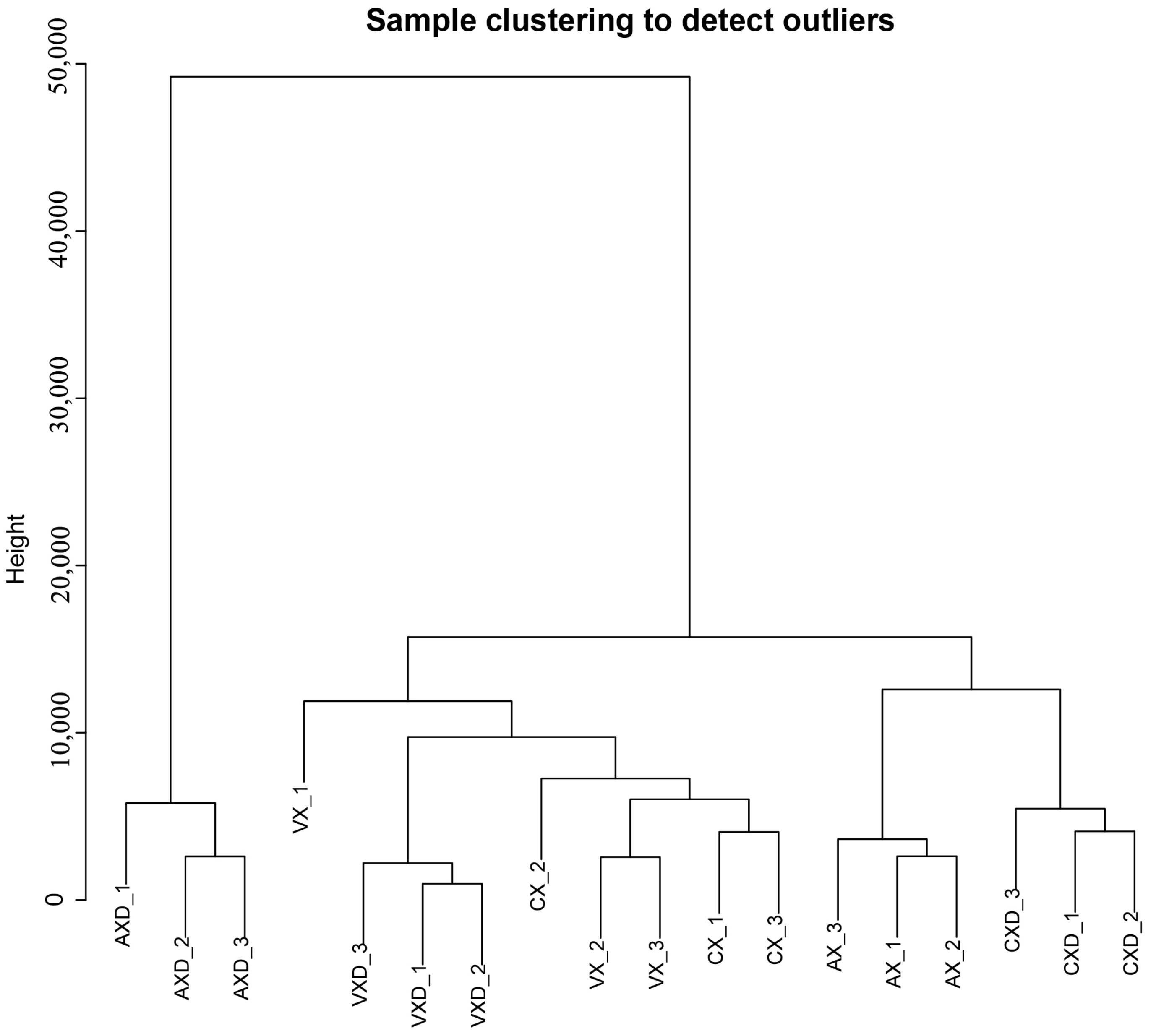

Supplement: Supplementary file 1 [file ijms-25-11575-s001.zip › ijms-3180441-supplementary/FigureS4.pdf]
