# Supplementary material for: Combined Transcriptome and Metabolome Analysis Reveals That Carbon Catabolite Repression Governs Growth and Pathogenicity in Verticillium dahliae
Source: Int J Mol Sci. 2024 Oct 28;25(21):11575. doi: 10.3390/ijms252111575 (PMC11546859; doi:10.3390/ijms252111575)
Supplement: Supplementary file 1 [file ijms-25-11575-s001.zip › ijms-3180441-supplementary/FigureS5.pdf]

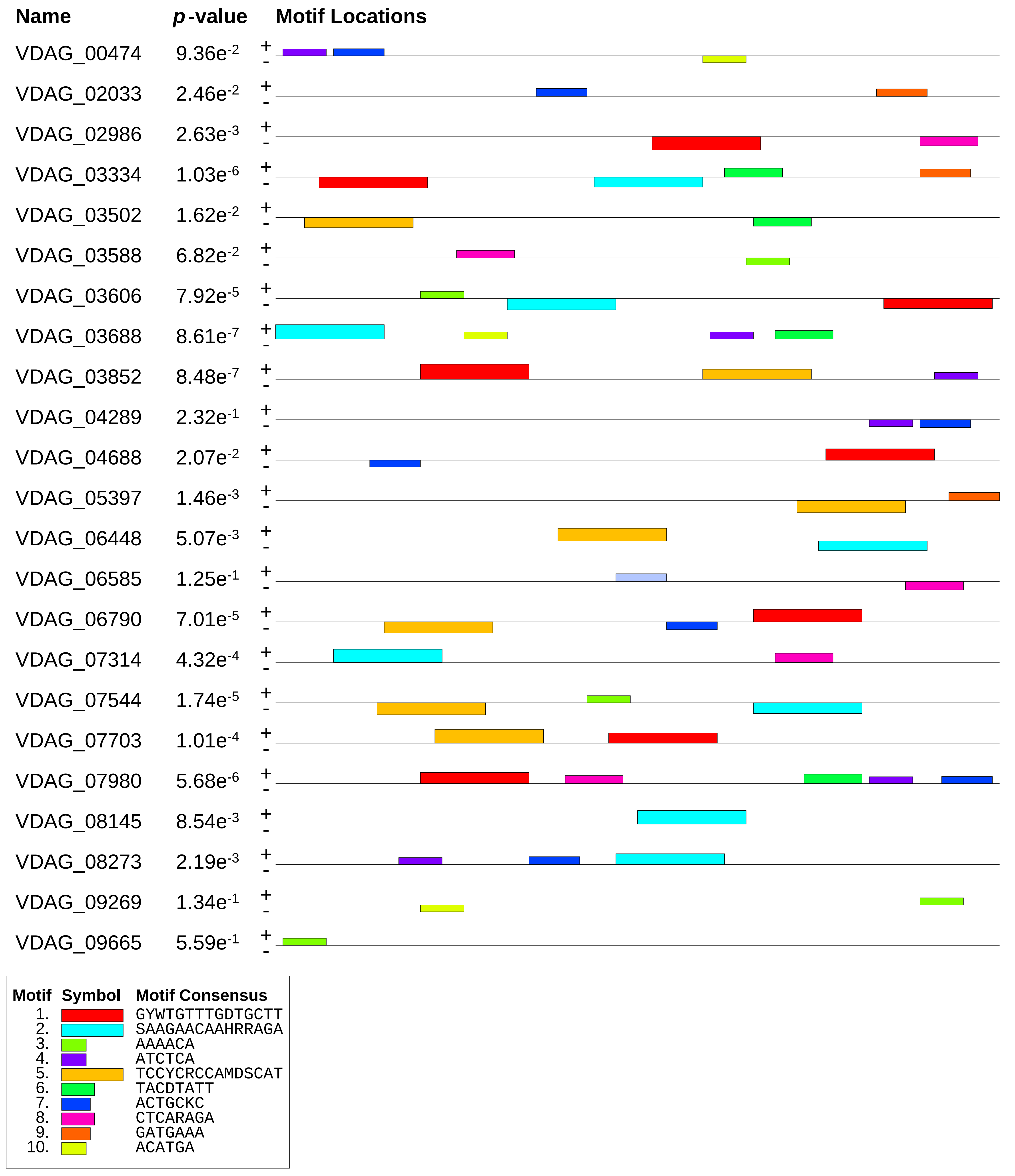
Name
p-value
Motif Locations
VDAG_00474 $9.36e^{-2}$
VDAG_02033 $2.46e^{-2}$
VDAG_02986 $2.63e^{-3}$
VDAG_03334 $1.03e^{-6}$
VDAG_03502 $1.62e^{-2}$
VDAG_03588 $6.82e^{-2}$
VDAG_03606 $7.92e^{-5}$
VDAG_03688 $8.61e^{-7}$
VDAG_03852 $8.48e^{-7}$
VDAG_04289 $2.32e^{-1}$
VDAG_04688 $2.07e^{-2}$
VDAG_05397 $1.46e^{-3}$
VDAG_06448 $5.07e^{-3}$
VDAG_06585 $1.25e^{-1}$
VDAG_06790 $7.01e^{-5}$
VDAG_07314 $4.32e^{-4}$
VDAG_07544 $1.74e^{-5}$
VDAG_07703 $1.01e^{-4}$
VDAG_07980 $5.68e^{-6}$
VDAG_08145 $8.54e^{-3}$
VDAG_08273 $2.19e^{-3}$
VDAG_09269 $1.34e^{-1}$
VDAG_09665 $5.59e^{-1}$
Motif Symbol Motif Consensus
1. GYWTGTTTGDTGCTT
2. SAAGAACAAHRRAGA
3. AAAACA
4. ATCTCA
5. TCCYCRCCAMDSCAT
6. TACDTATT
7. ACTGCKC
8. CTCARAGA
9. GATGAAA
10. ACATGA
