# Supplementary material for: Combined Transcriptome and Metabolome Analysis Reveals That Carbon Catabolite Repression Governs Growth and Pathogenicity in Verticillium dahliae
Source: Int J Mol Sci. 2024 Oct 28;25(21):11575. doi: 10.3390/ijms252111575 (PMC11546859; doi:10.3390/ijms252111575)
Supplement: Supplementary file 1 [file ijms-25-11575-s001.zip › ijms-3180441-supplementary/Supplementary figures.pdf]

Supplementary figures

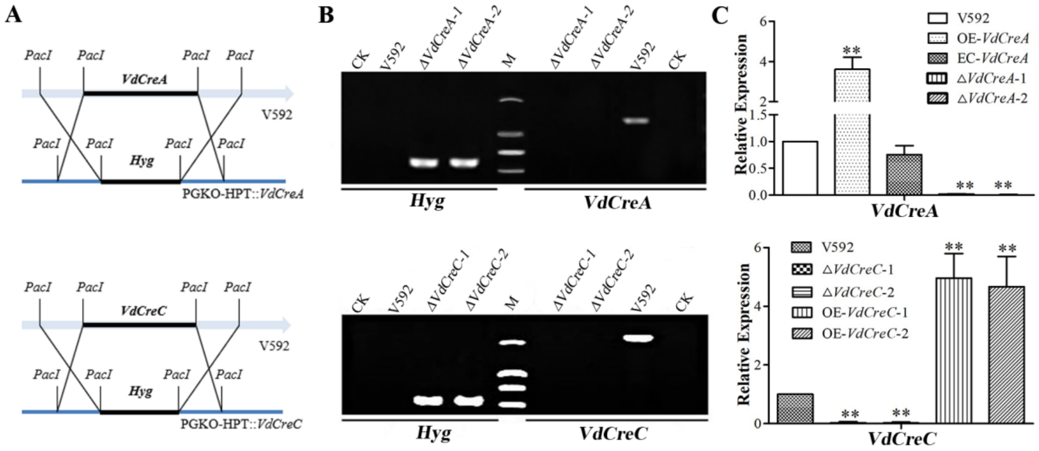

Figure S1. Knockouts of *VdCreA* and *VdCreC* in *V*. *dahliae* V592 strain. (A) Construction of *VdCreA* and *VdCreC* gene knockout vector. (B) Specific primers were used to detect *VdCreA* and *VdCreC* by PCR. (C) Confirmation of transcriptional expression of *VdCreA* and *VdCreC* by RT-qPCR analysis. ** represent significant difference at 0.01 probability level between knockout mutants, overexpression strains and wild-type strain V592 ($P < 0.01$).

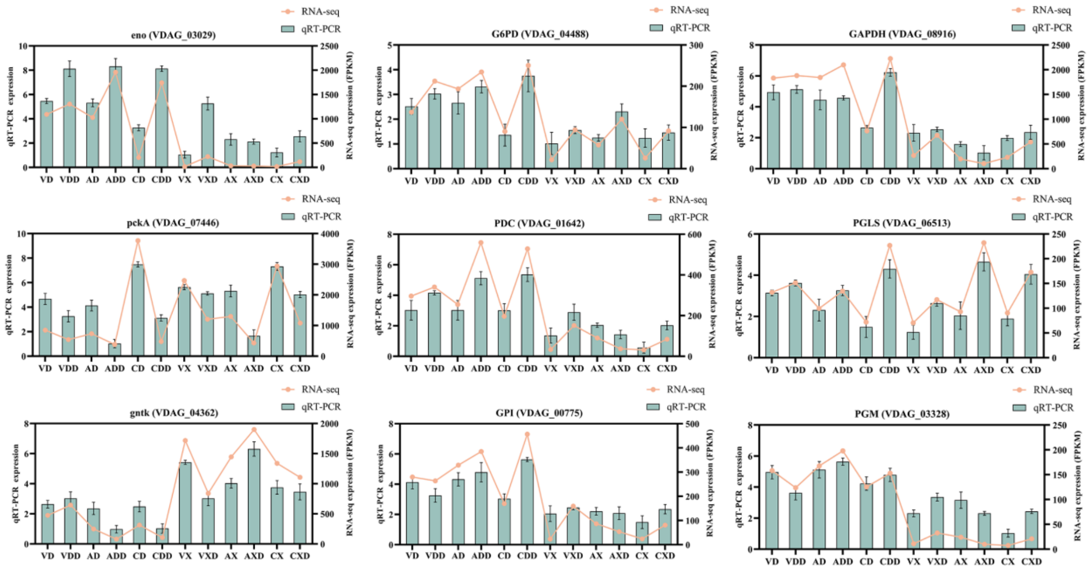

Figure S2. Analysis of RNA-seq data reliability.

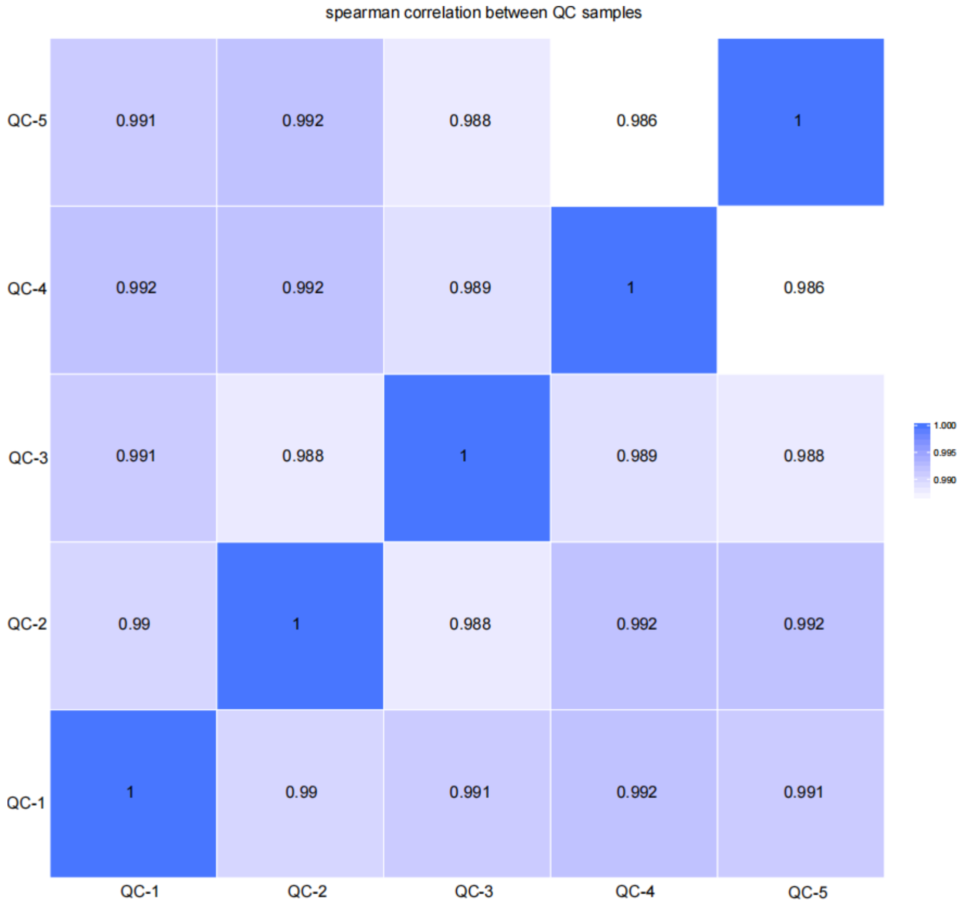

Figure S3. Expression correlation heat map of sample.

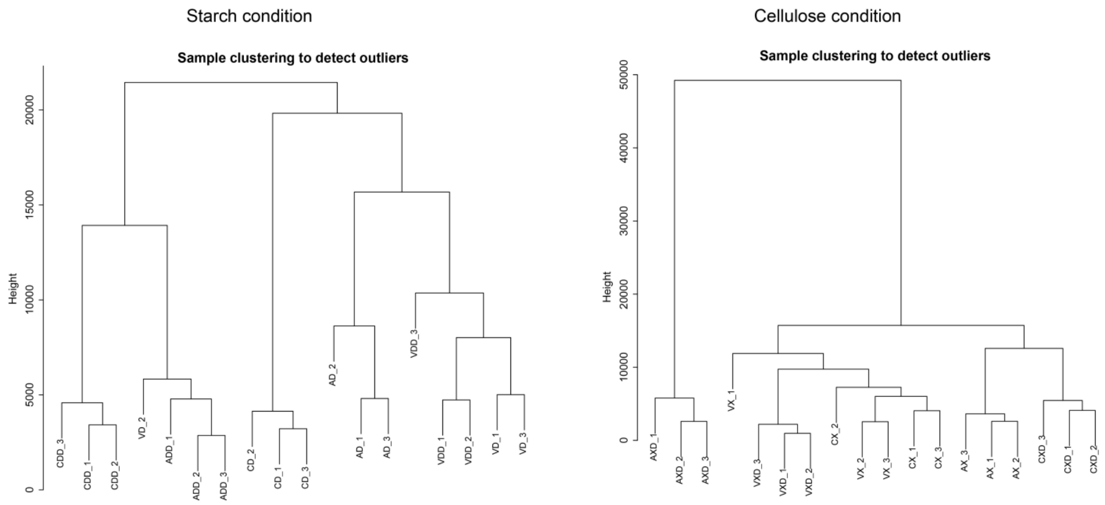

Figure S4. The phylogenetic tree was constructed based on expression levels of sample.

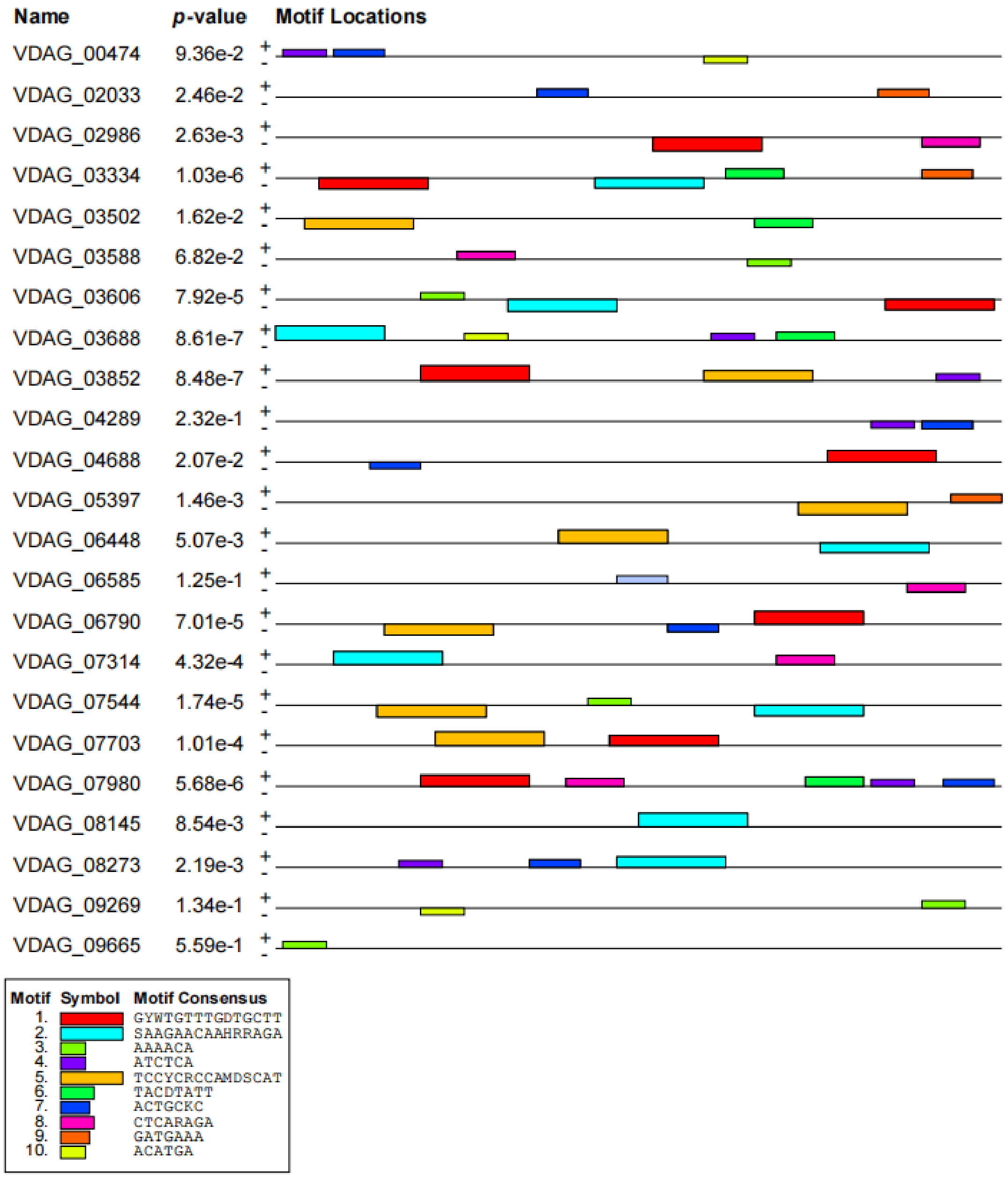

Figure S5. Conserved motif diagram of gene promoter co-expressed with *VdCreA*.

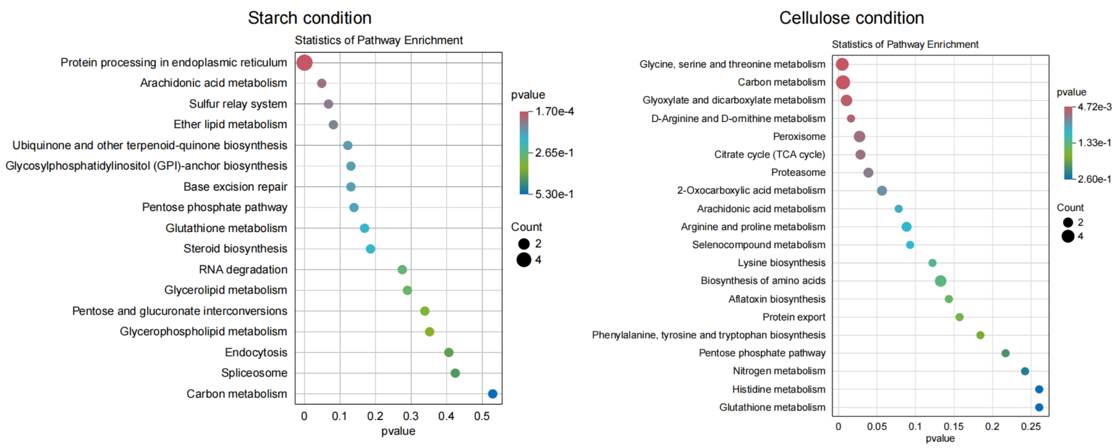

Figure S6. Module gene KEGG enrichment bubble diagram.
